# Xenon Impairs Reconsolidation of Fear Memories in a Rat Model of Post-Traumatic Stress Disorder (PTSD)

**DOI:** 10.1371/journal.pone.0106189

**Published:** 2014-08-27

**Authors:** Edward G. Meloni, Timothy E. Gillis, Jasmine Manoukian, Marc J. Kaufman

**Affiliations:** Department of Psychiatry, Harvard Medical School and McLean Hospital, Belmont, Massachusetts, United States of America; Sapienza University of Rome, Italy

## Abstract

Xenon (Xe) is a noble gas that has been developed for use in people as an inhalational anesthestic and a diagnostic imaging agent. Xe inhibits glutamatergic N-methyl-D-aspartate (NMDA) receptors involved in learning and memory and can affect synaptic plasticity in the amygdala and hippocampus, two brain areas known to play a role in fear conditioning models of post-traumatic stress disorder (PTSD). Because glutamate receptors also have been shown to play a role in fear memory reconsolidation – a state in which recalled memories become susceptible to modification – we examined whether Xe administered after fear memory reactivation could affect subsequent expression of fear-like behavior (freezing) in rats. Male Sprague-Dawley rats were trained for contextual and cued fear conditioning and the effects of inhaled Xe (25%, 1 hr) on fear memory reconsolidation were tested using conditioned freezing measured days or weeks after reactivation/Xe administration. Xe administration immediately after fear memory reactivation significantly reduced conditioned freezing when tested 48 h, 96 h or 18 d after reactivation/Xe administration. Xe did not affect freezing when treatment was delayed until 2 h after reactivation or when administered in the absence of fear memory reactivation. These data suggest that Xe substantially and persistently inhibits memory reconsolidation in a reactivation and time-dependent manner, that it could be used as a new research tool to characterize reconsolidation and other memory processes, and that it could be developed to treat people with PTSD and other disorders related to emotional memory.

## Introduction

Mitigation of persistent, intrusive, traumatic memories experienced by people with post-traumatic stress disorder (PTSD) remains a key therapeutic challenge [1]. Behavioral treatments such as extinction training – administered alone or in combination with cognitive-enhancing drugs (e.g. d-cycloserine) – attempt to inhibit underlying traumatic memories by facilitating a new set of learning contingencies, but often achieve limited success [2]. Another learning and memory phenomenon known as reconsolidation, a process by which reactivated (retrieved) memories temporarily enter a labile state (the reconsolidation window), has been studied to determine whether drug or behavioral interventions can prevent a traumatic memory trace from being re-incorporated back into the neural engram, inhibiting the memory [3]–[6]. Several chemical agents have been found to inhibit fear memory reconsolidation in animals [7] but unfortunately do not translate well to humans, limiting their clinical use. They either are toxic (e.g. protein synthesis inhibitors), induce unwanted side effects, are slow acting such that brain drug concentrations peak outside of the reconsolidation window, or are slowly eliminated such that they interfere with later onset memory processes including extinction [8]. A recent human study documented that a single electroconvulsive therapy (ECT) treatment administered to unipolar depressed subjects immediately after emotional memory reactivation disrupted reconsolidation, confirming that reconsolidation occurs in humans and that it can be inhibited by a brief treatment [9]. While ECT is indicated for therapeutic use in people with treatment-resistant major depression, it may not be a viable treatment for other clinical populations. Thus, there is a significant unmet need for a minimally invasive, safe and well-tolerated treatment that can be used clinically to inhibit fear memory reconsolidation in people with PTSD.

The noble gas xenon (Xe) inhibits glutamatergic N-methyl-D-aspartate (NMDA) receptors [10] known to play a role in memory reconsolidation [11]. Xe reduces NMDA-mediated synaptic currents and neuronal plasticity in the basolateral amygdala and CA1 region of the hippocampus [12], [13]; these brain areas are involved in Pavlovian fear conditioning, an animal model of PTSD used to elucidate learning and memory processes, including reconsolidation [14]–[16]. Xe already is used in humans at high concentration (>50%) as an anesthetic and at subsedative concentration (28%) as a diagnostic imaging agent; in both applications, Xe has excellent safety/side effect profiles and is well tolerated [17]–[19]. Further, NMDA receptor glycine antagonists like Xe [10] do not appear to have significant abuse liability and do not induce psychosis [20], [21], consistent with clinical experience [18], [19]. Thus, Xe has a number of favorable properties that might be beneficial for treating fear memory disorders. As fear memory reconsolidation is an “evolutionarily conserved memory-update mechanism” [5], we evaluated in rats whether administering a subsedative concentration of Xe (maximum concentration 25%, 1 h) via inhalation following conditioned fear memory reactivation could reduce subsequent expression of fear-like behavior. Here, we report that Xe impaired reconsolidation of fear memory demonstrated as a reduction in conditioned freezing, a behavioral readout used to measure fear in animals.

## Methods and Materials

### Experimental subjects

Male Sprague-Dawley rats (Charles River; Raleigh, NC) weighing 350–375 g were pair-housed in plastic Nalgene rat cages and acclimated to the main animal vivarium for two weeks before being randomly assigned to different treatment groups (below). Rats were maintained on 12/12 h light dark cycles and food and water were provided *ad libitum*. Experiments were performed from 10 a.m. to 3 p.m. All animal handling was limited to the time required to transport and place animals in the fear-conditioning chambers and air/xenon exposure chambers (i.e., no pre-study handling acclimation was used). The sample size was determined in concordance with our previous work examining reconsolidation mechanisms using the conditioned- freezing behavioral assay [22]. All animal procedures were approved by McLean Hospital's Institutional Animal Care and Use Committee (Office of Laboratory Animal Welfare Assurance number A3685–01) in accordance with the National Institute of Health *Guide for the Care and Use of Laboratory Animals (8^th^ Edition)*.

### Fear-conditioning apparatus

Conditioning and testing were conducted in four identical 19×9×14 cm Plexiglas behavioral chambers contained in a sound-attenuating cubicle (Med-Associates, Georgia VT). On the training day, rats were placed in chambers and after 2 min received two pairings of a 30 s, 5 kHz, 75 dB tone (conditioned stimulus; CS) co-terminating with a 0.6 mA, 0.5 s footshock (unconditioned stimulus; US) delivered through the floorbars of the chamber. Shock reactivity (cage movement in response to shock delivery) was measured after each training trial by an accelerometer at the base of the cage. Accelerometer analog output was amplified and digitized on a scale of 0–20 units by an analog-to-digital card interfaced with a PC computer (Med-Associates). Animals with shock reactivity levels <3 units (averaged across two training trials) were excluded as this can be used as an indicator of the strength of conditioning (i.e. weak shock reactivity) [23]; a total of 5 out of 99 animals were excluded based on this criteria. The intertrial interval of CS-US pairings was 30 s. After an additional 30 s in the chamber, animals were returned to their home cages. Memory was reactivated (Reactivation) 24 h after training by returning animals to testing chambers and after 2 min animals were exposed to the tone CS (5 kHz, 75 dB) for 60 s. Post-reactivation long-term memory (PR-LTM) was subsequently probed at 48 h (PR-LTM1), 96 h (PR-LTM2) or 18 d (PR-LTM3) using Reactivation day procedures. Freezing behavior was video-recorded on each day and scored by an experimenter blind to treatment conditions. Percent freezing was calculated as the % total time that animals remained immobile (frozen), other than breathing, during the first 2 min of re-exposure to the chamber (Context) and during 60 s CS presentation (Tone).

### Xenon exposure apparatus

**Figure 1:**
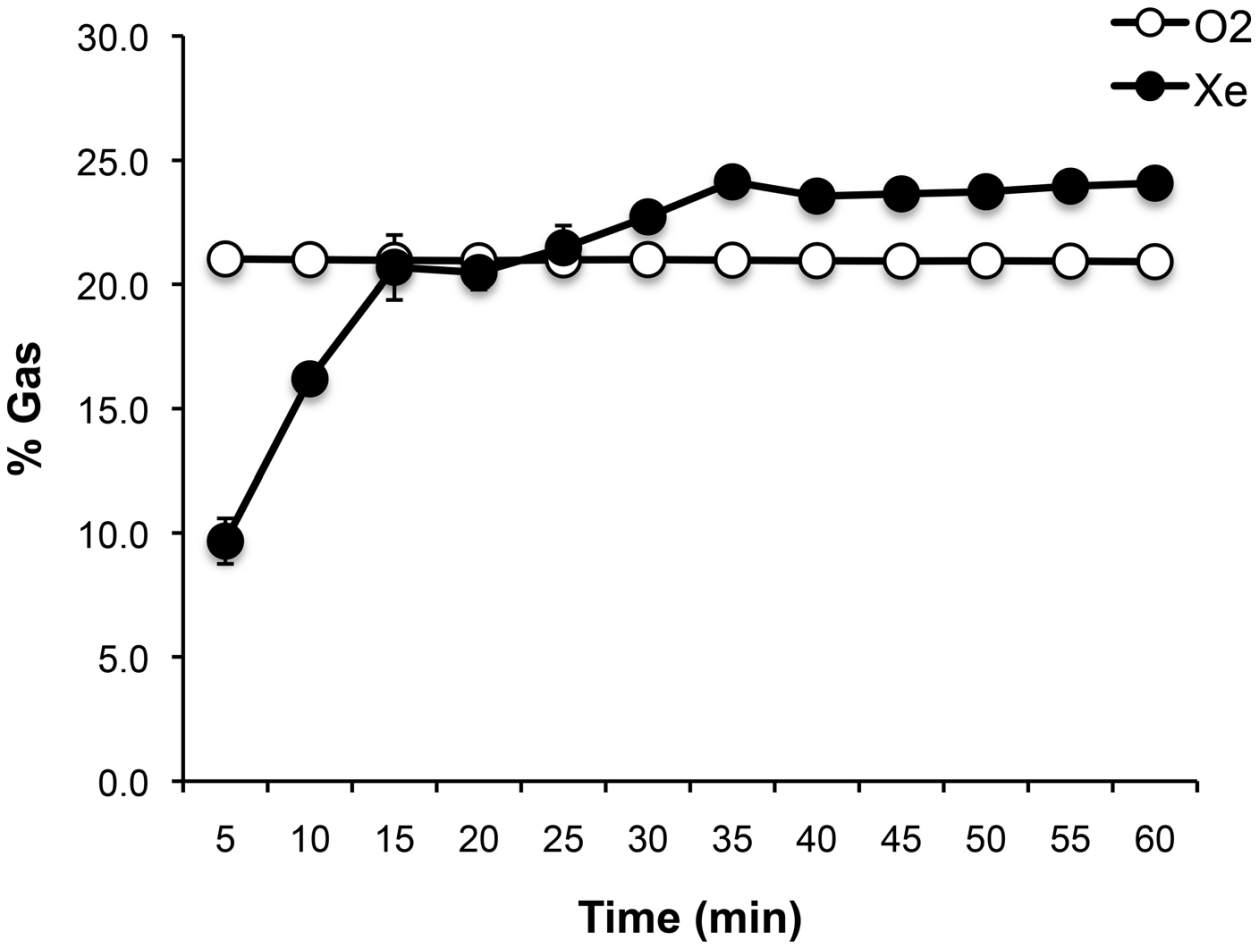
Xenon- (Xe) and oxygen (O2) gas concentration time course averaged across all exposures in this study. Percent Xe reflects exposure chamber atmospheric Xe concentration along with oxygen (maintained at 20.9%) and balanced with nitrogen. The rate of Xe delivery was approximately 2.5 liters per minute (supplied as 100% Xe from an external tank) and continuously mixed with chamber atmosphere by circulating fans to reach a maximum preset concentration of 25%. Data included in the figure are from 14 total Xe exposures (Fig. 2B &C, *n* = 3 runs; Fig. 2D & E, *n* = 3 runs; Fig. 2F & G, *n* = 2 runs; Fig. 3 B & C, *n* = 3 runs after Reactivation test and 3 runs after PR-LTM1). Data are shown as mean ± s.e.m.

A custom-built system (Air Products and Chemicals, Inc.; Bethlehem, PA – APCI) was used to expose animals to 25% xenon (Xe) gas (Praxair, Inc.; Danbury, CT). The apparatus consisted of a 30×24×16 in. sealable Plexiglas chamber capable of housing two modified Nalgene rat cages (perforated along all sides to facilitate gas exchange) for exposure of up to four rats at a time (2 rats/cage). The delivery (rate and concentration) both of Xe and supplemental oxygen (as needed to maintain 20.9% concentration; **Figure 1**) was regulated by PC-interfaced mass-flow controllers using custom-designed software (APCI). Xenon, oxygen, carbon dioxide, pressure, temperature and humidity were all monitored by sensors in the system and compensated as needed by the internal control system and supporting equipment to maintain set levels. An identical system was used for air exposures except that only normal room air was supplied. Both the Xe and air-exposure apparatuses were located in a dedicated animal quarantine bay apart from the main vivarium but maintained under the same environmental conditions.

### Experimental procedures

After two-weeks acclimation in the main vivarium, pairs of rats either were left in the rat housing room (Normal exposure group) or re-located to the Xe (Xenon group) or Air (Air group) chambers for further acclimation (1 week) to experimental-housing settings. Rats then were trained for contextual and cued fear conditioning using procedures adapted from Phillips and LeDoux [24]. Accordingly, this allowed us to evaluate the expression of conditioned freezing in the presence of a conditioned stimulus (CS, a tone) and the context (the conditioning/test chamber) present during the training (CS pairing with shock, the unconditioned stimulus; US), and to examine effects of Xe administered after memory reactivation on both components (freezing to context and tone).

**Figure 2:**
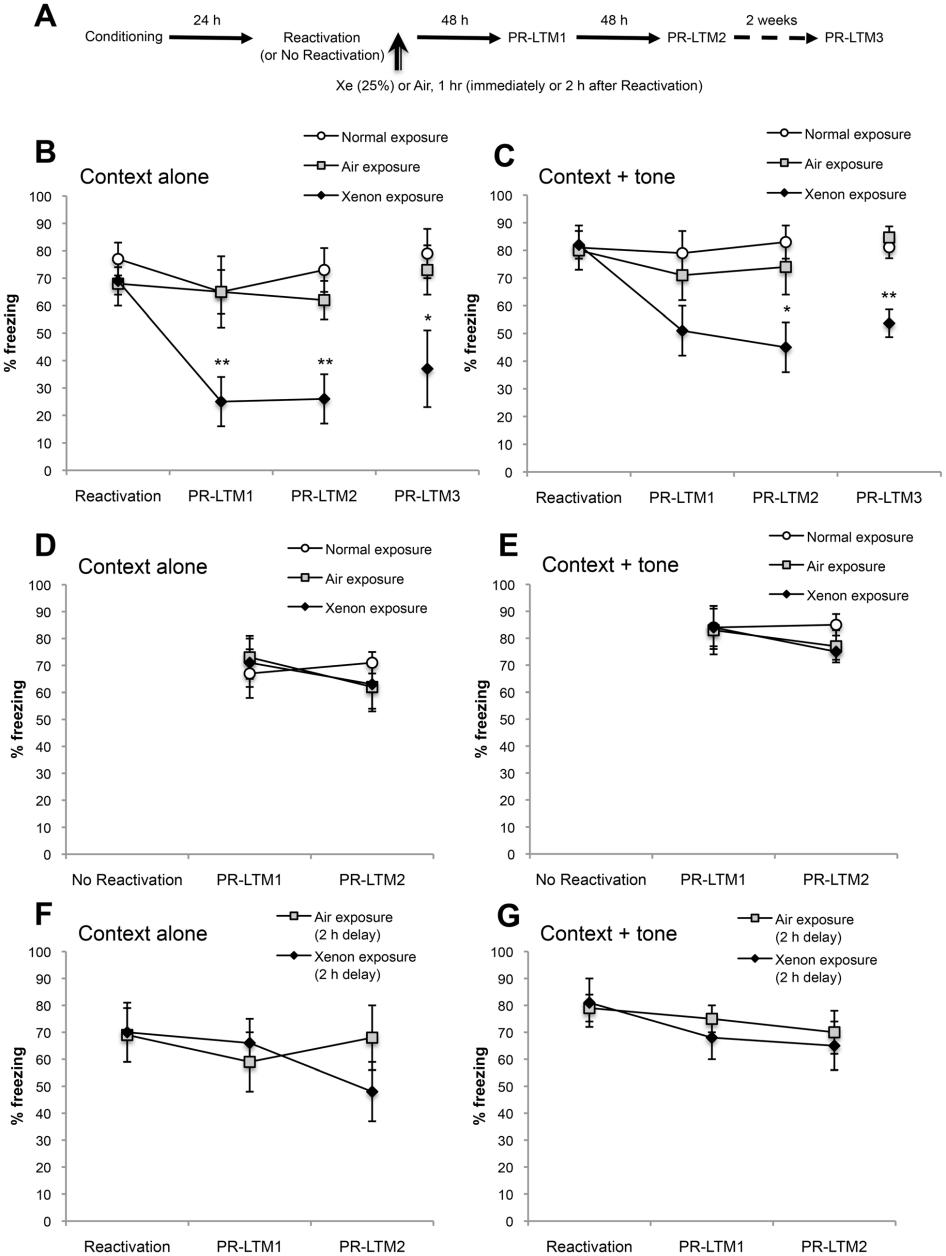
Xenon exposure impairs reconsolidation and reduces conditioned freezing in a reactivation- and time-dependent manner. (**A**) Schematic of the experimental design. Twenty-four h after fear conditioning, fear memories either were or were not reactivated and rats were exposed either to Xe (25%) or Air for 1 h beginning either immediately or after a 2 h delay. An additional control group housed in the regular main rat vivarium (Normal exposure) also was included in some studies to control for any potential effects of housing in the exposure chambers. Post-reactivation long-term memory (PR-LTM) was subsequently probed 48 h (PR-LTM1) and 96 h (PR-LTM2) later. A subset of rats (n = 6–7/group) were further tested for spontaneous recovery of freezing by testing 18 d after Reactivation (PR-LTM3). (**B & C**) Percent freezing to context alone and context + tone (respectively) in animals exposed to Xe (25%) or Air immediately after Reactivation. **P<0.005; *P<0.05 compared to Air exposure. Normal exposure, *n* = 8; Air exposure, *n* = 11; Xe exposure, *n* = 11. (**D & E**) Percent freezing to context alone and context + tone in rats not receiving a Reactivation test. Normal exposure, *n* = 8; Air exposure, *n* = 11; Xe exposure, *n* = 11. (**F & G**) Percent freezing to context alone and context + tone in rats exposed either to Xe (25%) or Air beginning 2 h after Reactivation. Air exposure, *n* = 7; Xe exposure, *n* = 8. Data are shown as mean ± s.e.m.

The timeline of procedures used for fear conditioning, testing and Xe exposure is illustrated in **Figures 2A** **&** **3A**. On Day 1, rats were fear conditioned as described above. Twenty-four hours later, immediately following reactivation testing, animals either were placed in Xe or Air-exposure chambers, lids were sealed, and animals were exposed to Xe (25%) or room air for 1 h. After 1 h, Xe was completely scavenged by the Xe-exposure system and chamber lids were opened to normal room air exposure for the duration of the study.

**Figure 3:**
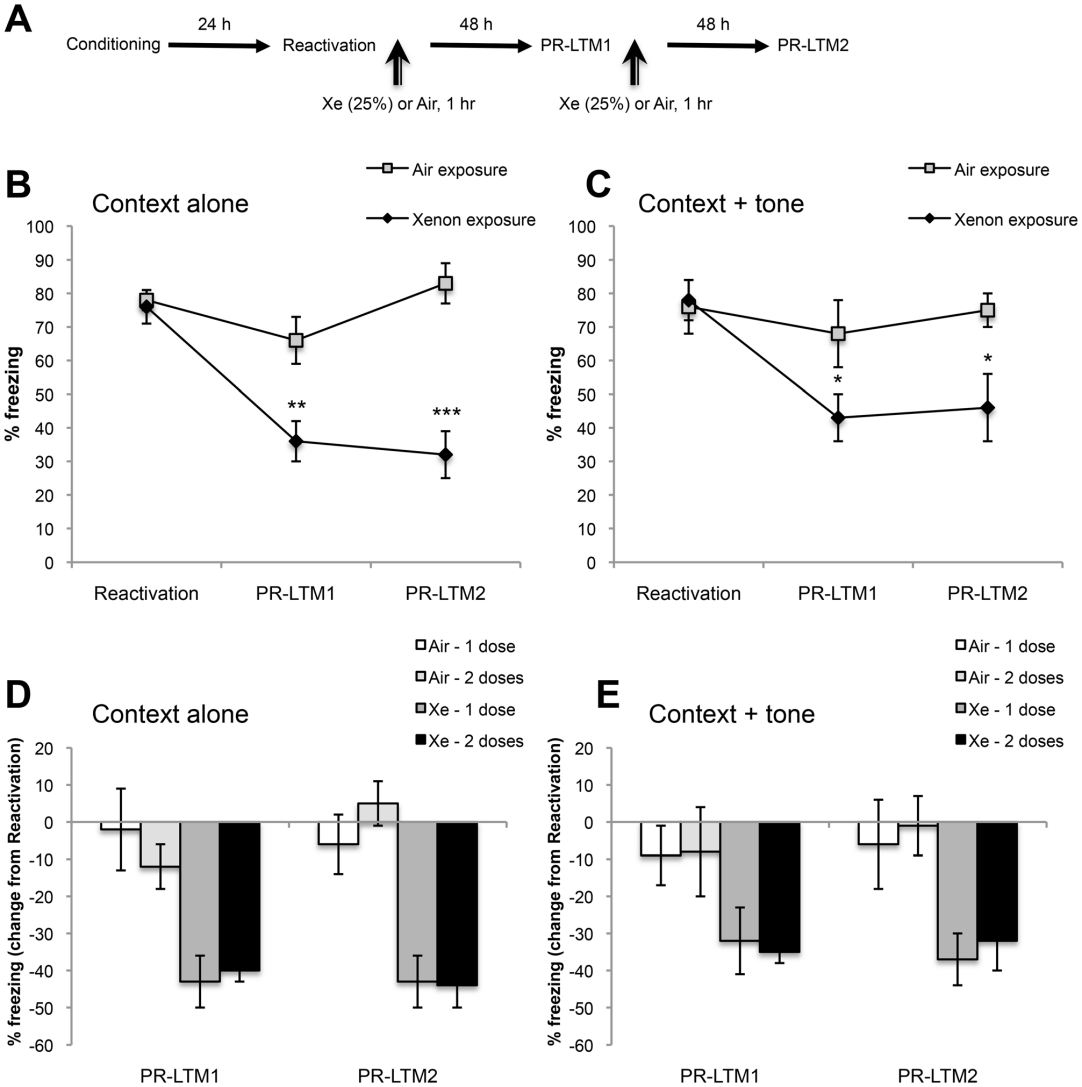
Multiple Xe exposures after fear memory reactivations do not enhance amnestic effects on conditioned freezing. (**A**) Schematic of the experimental design for multiple Xe-exposure treatment. In addition to administering Xe (25%) or Air for 1 h after Reactivation, animals were exposed a second time to Xe (25%) or Air for 1 h immediately after PR-LTM1 and freezing was again probed 48 h later, (PR-LTM2). (**B & C**) Percent freezing to context alone and context + tone (respectively) in animals exposed to Xe (25%) or Air for 1 hr immediately after Reactivation and PR-LTM1. (**D & E**) Normalized freezing data to context and tone. Data are expressed as % differences from the first Reactivation test day in order to compare the effects of multiple Xe exposures. A second Xe exposure did not alter freezing either to context alone or context + tone at PR-LTM2 compared to PR-LTM1. ***P<0.0005; **P<0.005; *P<0.05; Air–1 exposure, *n* = 11; Air–2 exposures, *n* = 9; Xe–1 exposure, *n* = 11; Xe–2 exposures, *n* = 10. Data are shown as mean ± s.e.m.

A second set of animals was trained as described above but did not receive a reactivation test 24 h later. Instead, at this time-point, animals were exposed either to 25% Xe or Air for 1 h to determine whether Xe must be paired with memory reactivation for it to affect memory reconsolidation.

A third set of animals was trained as described above, underwent reactivation 24 hours later, and were exposed either to 25% Xe or Air (1 h, both types of exposure) beginning 2 h after the reactivation test, to determine whether delayed Xe exposure affected freezing at PR-LTM1 and PR-LTM2.

A fourth set of animals was trained as described above and exposed either to 25% Xe or Air for 1 h twice; immediately after reactivation and again after reactivation during PR-LTM1 testing, to determine whether multiple Xe exposures enhance reconsolidation blockade.

### Statistics

Two-way ANOVAs for treatment group (between-subjects) × test day (within-subjects) comparisons were performed. Comparisons between treatment groups for the PR-LTM3 test day (long term test for spontaneous recovery), in a subset of animals, were performed using one-way ANOVA. For measurements yielding significant main effect, subsequent multiple pairwise comparisons were made using Dunn's test. All reported *t* tests are two-sided measures.

## Results

Rats exposed to Xe (25%, 1 h) immediately after fear memory reactivation exhibited a significant reduction of freezing when tested 48 and 96 h after reactivation (PR-LTM1 and PR-LTM2, respectively) compared to air-exposed controls (**Fig. 2B & 2C**). Main effects: context alone (treatment group: F_2,27_ = 6.31, P = 0.006; test day: F_2,54_ = 10.41, P = 0.0001; interaction: F_4,54_ = 4.99, P = 0.002); context + tone (treatment group: F_2,27_ = 3.27, P = 0.05; test day: F_2,54_ = 4.41, P = 0.02; interaction: F_4,54_ = 2.63, P = 0.04). Xe-exposed rats exhibited a trend for reduced freezing in the context + tone condition 48 h after reactivation (P = 0.06 compared to Air-exposure; P = 0.02 compared to main vivarium (Normal)- exposure); the reduction attained statistical significance versus Air-exposed controls when reassessed 96 h after reactivation (i.e. at PR-LTM2; **Fig. 2C**). In a separate cohort treated identically to the first cohort up through the PR-LTM1 test, the Xe effect on freezing to the context alone finding was replicated while Xe significantly reduced freezing to the context + tone at the first post-reactivation test (PR-LTM1; **Fig. 3B & 3C**). When all observations of Xe effects on freezing at PR-LTM1 were pooled from these two independent experiments (Air-exposure, *n* = 20; Xe-exposure, *n* = 21) there was a highly significant reduction in freezing both to context alone (*t*_39_ = 4.63, P<0.0001) and context + tone (*t*_39_ = 2.16, P<0.01) compared to air-exposed controls. Freezing at PR-LTM1 and PR-LTM2 did not significantly differ.

In order to examine whether the amnestic effects of xenon were long-lasting, a subset of animals from each treatment group was further tested 18 days after the Reactivation test. On this test day (PR-LTM3), freezing both to the context alone and to context + tone was significantly reduced in Xe-exposed rats compared to control groups, indicating a lack of spontaneous recovery of the fear memory over time. Main effects: context alone (treatment group: F_2,16_ = 3.76, P = 0.04); context + tone (treatment group: F_2,16_ = 10.93, P = 0.001).

**Figure 2D & E** show that post-reactivation freezing to context alone and context + tone (respectively) in rats that were exposed to Xe but that did not receive a Reactivation test was not significantly different from controls (no significant main effects). **Figure 2F & G** show that post-reactivation freezing to context alone and context + tone (respectively) in rats exposed to Xe 2 h after Reactivation was not significantly different from controls (no significant treatment effects). Together, these data indicate that Xe was only effective at reducing long-term expression of freezing when administered in conjunction with memory reactivation and within the putative reconsolidation window.

**Figure 3B & C** show that multiple Xe exposures after fear memory reactivations do not further enhance the amnestic effects of Xe on conditioned freezing. Main effects: Percent freezing to context alone (treatment group: F_1,17_ = 14.9, P = 0.001; test day: F_2,34_ = 25.1, P<0.0001; interaction: F_2,34_ = 20.1, P<0.0001); percent freezing to context + tone (treatment group: F_1,17_ = 3.6, P = 0.07; test day: F_2,34_ = 6.9, P = 0.003; interaction: F_2,34_ = 4.2, P = 0.02). **Figure 3D & E** show normalized freezing data to context alone and context + tone. Data are expressed as % differences from the first Reactivation test day in order to compare the effects of multiple Xe exposures. As shown, a second Xe exposure did not alter freezing either to context alone or context + tone at PR-LTM2 compared to PR-LTM1 (no significant differences between PR-LTM1 and PR-LTM2).

## Discussion

Here, we report for the first time, that inhaled administration of a subsedative concentration of Xe gas substantially and persistently inhibits a long-term fear memory, but only after memory reactivation and when administered within the putative reconsolidation window [25]. NMDA receptor dynamics appear to play key roles in both the destabilization and reconsolidation of memory [11], [26], [27] and Xe's rapid inhibition of these receptors post-reactivation could mediate the effects we observed. Xe directly reduces NMDA-mediated synaptic currents and affects neuronal plasticity in the basolateral amygdala and CA1 region of the hippocampus [12], [13], brain areas known to play a role in fear conditioning and which have been implicated in the pathophysiology of PTSD [28], [29]. Xe also may indirectly reduce NMDA receptor function by inhibiting the enzyme tissue plasminogen activator (tPA) [30]. tPA increases NMDA receptor activity by proteolytically cleaving the NR1 subunit amino terminal domain [31]. Although tPA is best known as a clot-busting drug used in acute stroke patients, tPA is released from dendrites during synaptic activity [32], especially during high frequency stimulation [33], tPA acts as a gliotransmitter [34], and tPA participates in synaptic plasticity and learning and memory processes including fear conditioning [35], [36]. Xe also had been reported to affect AMPA receptors [12] shown to play a role in memory reconsolidation [37], [38]. Collectively, Xe's direct and indirect inhibition of NMDA and AMPA receptor function may underlie its ability to impair fear memory reconsolidation.

Other targets of Xe also could mediate the effects we observed. For example, Xe has also been shown to have differential effects on excitatory and inhibitory ligand-gated ion channels; Xe reduces current through alpha4 beta2 (α4β2) nicotinic acetylcholine receptor-gated channels and increases current through glycine and GABA_A_ receptor-gated channels [39]. Xe also targets other proteins known to play a role in contextual fear memory including alpha7 (α7) nicotinic acetylcholine receptors [40], [41] and ATP-dependent potassium (Kir6.2) channels [42], [43], and targets TREK-1 channels [44]. At this time, we cannot conclude which targets of Xe mediate its inhibition of fear memory reconsolidation, which is a limiting factor of this study. Future studies are planned, however, using selective agonists and antagonists of these and other receptors and proteins, to characterize the pharmacology and mechanism of action of Xe's effects on reconsolidation.

In our analysis of the pooled sample from all Xe-treated rats (*n* = 21) we found a within-subjects difference in Xe's effects at PR-LTM1 on freezing to context alone and context + tone whereby freezing in the presence of the tone was less sensitive to Xe (*t*_20_ = 3.72, P<0.005). These data suggest that Xe's amnestic effects may be stronger for context- versus cue-induced freezing. This may reflect a stronger effect of Xe on the hippocampus than the amygdala, which play different roles in context and cued-fear conditioning [15], [24]. This differential effect could be related to Xe's apparently greater inhibition of hippocampal versus amygdala excitatory postsynaptic currents [12], [13]. Interestingly, other inhaled anesthetic agents, such as halothane, isoflurane, and nitrous oxide, which can also affect learning and memory and have amnestic effects, can alter hippocampal theta rhythms [45] which have been shown to contribute to reconsolidation of contextual fear memory by virtue of its synchronization with the amygdala [46]. Hence, a preferential action of Xe on hippocampal ensemble activities could account for the strong amnestic effect upon re-exposure to the conditioning context (hippocampal dependent), but which was reduced when the animal was then presented with a discrete cue (amygdala dependent) within the conditioning context. Clearly, a limitation of the current study is that animals were not tested for cue-induced freezing in a different context than that used for fear-conditioning, and that Xe was tested in only one fear-conditioning paradigm. Our intention in these initial studies was to elucidate the basic phenomenon using a paradigm similar to that used in the seminal studies of Phillips and LeDoux [24], which established a differential role for the hippocampus and amygdala in context versus cued fear-conditioning. Future studies investigating Xe's effects on reconsolidation for context- and cue-induced freezing, including freezing elicited in a different context are planned, as well as studies involving different fear conditioning paradigms.

As described in Tronson and Taylor [3], a number of control protocols can be employed to demonstrate that a specific treatment affects reconsolidation. The data presented in this report include several of these important comparison groups. First, we demonstrate that rats exposed to 25% Xe for 1 hour in the absence of fear memory reactivation exhibited no differences in freezing to context or tone versus air-exposed controls (**Fig. 2D & E**). These data suggest that the effects of Xe on reconsolidation and impairment of long-term fear memory are not due to non-specific effects of Xe gas inhalation, but that Xe's effects likely are having a direct effect on brain mechanisms engaged only after the fear memory is recalled. Second, when Xe administration was delayed until 2 hours after fear memory reactivation, a time point expected to be outside of the reconsolidation window for NMDA antagonists [25], Xe was ineffective at reducing freezing (**Fig. 2F & G**). Together, these results suggest that 25% Xe inhibits fear memory reconsolidation only after fear memory reactivation and only when administered within the reconsolidation window. These findings along with our data showing a lack of spontaneous recovery – a traditional test used to examine the enduring amnestic effect of a treatment [47] – document that Xe satisfies several requirements of a demonstrable reconsolidation-blocking agent.

Lastly, we examined whether a second 25% Xe exposure for 1 hour immediately after the PR-LTM1 test (which is, itself, another memory reactivation), could further impair reconsolidation. The second Xe exposure did not further affect freezing to context alone or context + tone (**Fig. 3B & 3C**) when compared with Xe administered only after first reactivation (**Fig. 3D & 3E**). Possible interpretations of this finding include that the Xe-sensitive component of reconsolidation may have a threshold for its amnestic capacity after blockade and/or that residual fear maintenance may occur by Xe-insensitive mechanisms. A recent study reported that a transition state may develop after multiple unreinforced CS exposures reflecting a dynamic shift from reconsolidation to extinction processes, during which NMDA receptor antagonists lose their ability to affect reconsolidation or extinction [48]. As both processes are NMDA-receptor dependent [11], it is possible that a first treatment with Xe and the ensuing amnestic effect may shift the reconsolidation-extinction boundaries to limit the effectiveness of a second treatment. Xe's rapid on-off kinetics [49] may facilitate its use both in animals and humans as a temporally precise tool to help characterize such transition states and other dynamic memory processes. Given that the timing of interventions aimed at blocking reconsolidation or enhancing extinction may significantly affect treatment outcomes [11], [48], Xe’s rapid kinetics also may enable temporally optimized treatment regimens.

In summary, we report in an animal model of PTSD that 25% Xe administered within the reconsolidation window after fear memory reactivation substantially reduced subsequent fear memory expression. This anxiolytic-like effect in rats has translational application to current clinical research aimed at modulating memory processes as a therapy for fear and anxiety disorders [50]–[53]. People with PTSD experience intrusive, persistent traumatic memories [54], impaired fear memory extinction [55], and may be locked in reconsolidation mode [56]. Given that fear memory reconsolidation is “evolutionarily conserved” [5] and that subsedative Xe inhalation is associated with well-established excellent safety and side-effect profiles in humans [18], [19] Xe appears to have potential for rapid development as a pharmacotherapy to inhibit traumatic memory reconsolidation in PTSD patients, and possibly treat other conditions involving reconsolidation, including addiction disorders [56], [57].
